# Effect of vaccine reminder and tracker bracelets on routine childhood immunization coverage and timeliness in urban Pakistan: protocol for a randomized controlled trial

**DOI:** 10.1186/s12889-019-7667-3

**Published:** 2019-10-30

**Authors:** Danya Arif Siddiqi, Mehr Munir, Mubarak Taighoon Shah, Aamir Javed Khan, Subhash Chandir

**Affiliations:** 1 IRD Pakistan, 4th Floor, Woodcraft Building, Plot 3 & 3-A, Sector 47, Korangi Creek Road, Karachi, Pakistan; 2 IRD Global, 583 Orchard Road, #06-01 Forum, 238884, Singapore; 3 Harvard Medical School Center for Global Health Delivery, Building 14, Dubai Healthcare City, Dubai, United Arab Emirates

**Keywords:** Immunization coverage and timeliness, Silicone bracelets, Immunization reminders

## Abstract

**Background:**

Inability to track children’s vaccination history coupled with parents’ lack of awareness of vaccination due dates compounds the problem of low immunization coverage and timeliness in developing countries. Traditional Reminder/Recall (RR) interventions such as paper-based immunization cards or mHealth based platforms do not yield optimal results in resource-constrained settings. There is thus a need for a low-cost intervention that can simultaneously stimulate demand and track immunization history to help reduce drop-outs and improve immunization coverage and timeliness. The objective of this study is to evaluate the impact of low-cost vaccine reminder and tracker bracelets for improving routine childhood immunization coverage and timeliness in Pakistani children under 2 years of age.

**Methods:**

The study is an individually randomized, three-arm parallel Randomized Controlled Trial with two intervention groups and one control group. Infants in the two intervention groups will be given two different types of silicone bracelets at the time of recruitment, while infants in the control group will not receive any intervention. The two types of bracelets consist of symbols and/or numbers to denote the EPI vaccination schedule and each time the child will come for vaccination, the study staff will perforate a hole in the appropriate symbol to denote vaccine administration. Therefore, by looking at the bracelet, caregivers will be able to see how many vaccines have been received. Our primary outcome measure is the increase in coverage and timeliness of Pentavalent-3/PCV-3/Polio-3 and Measles-1 vaccine in the intervention versus control groups. A total of 1446 participants will be recruited from 4 Expanded Program on Immunization (EPI) centers in Landhi Town, Karachi. Each enrolled child will be followed up till the Measles-1 vaccine is administered, or till eleven months have elapsed since enrolment.

**Discussion:**

Participant recruitment commenced on July 19, 2017, and was completed on October 10, 2017. Proposed duration of the study is 18 months and expected end date is December 1, 2018. This study constitutes one of the first attempts to rigorously evaluate an innovative, low-cost vaccine reminder bracelet.

**Trial registration:**

ClinicalTrials.gov NCT03310762. Retrospectively Registered on October 16, 2017.

## Background

Immunization is one of the most cost-effective ways of preventing childhood disability, morbidity and mortality caused by infectious diseases. Despite the substantial advances achieved by governments in establishing and maintaining national immunization systems, immunization coverage is low and delayed, and completion rates are poor in many developing countries. It is estimated that yearly, about 18.7 million infants worldwide are not reached by basic immunization services [1], and over 2 million children die from vaccine-preventable diseases annually [2]. Of the 19.5 million children globally who did not receive 3 doses of DTP vaccine in 2016, around 1.4 million (7%) lived in Pakistan [3] where coverage of Pentavalent 3 remains modest, ranging from below 20 to 80% across regions [4]. While increasing coverage of vaccines remains a priority, timely administration of vaccines remains equally important for success of immunization programs. Increased compliance to vaccine timeliness ensures that children are protected prior to exposure [5] and impacts morbidity through improving population immunity and potential spread of communicable diseases particularly in the form of disease outbreaks [6]. Children who are not vaccinated timely are much less likely to be fully vaccinated at later time due to further delays in subsequent vaccines, a finding corroborated from studies both in developing [5] and developed countries [7].

An increasingly recognized global challenge that leads to stagnating or declining coverage rates and poor timeliness is low uptake and demand for immunization services at the community level. Although the necessity of stimulating caregiver demand for childhood vaccines has been established through extensive research [8–11] developing country governments continue to focus almost exclusively on improving supply. As a result, they often fail to draw on innovative, low-cost public health campaigns to encourage end-users to seek and avail affordable health care. There is a need to develop and leverage demand-side interventions to transform communities from passive to active recipients of immunization services, thus increasing uptake.

This study addresses the gap of low demand for and limited community uptake of immunization services by introducing a simple tool that empowers caregivers by enhancing their awareness of the routine immunization schedule. Although some caregivers may resist immunization, many simply fail to complete a cycle because they are unaware of due dates and required number of vaccination visits, procrastinate, forget [12], or let other priorities get in the way [13]. Investigating effective and innovative ways of improving uptake of routine immunization, this study focuses on one potential channel to stimulate end-user demand: silicone bracelets for children that can serve as effective reminders for parents for timely immunization of their child. These bracelets can potentially save thousands of lives by giving children a chance to complete their immunizations timely. Furthermore, the bracelets can support Pakistan’s vaccine delivery system by encouraging mothers to return on-time for vaccinations without expending resources on campaigns that involve vaccinators combing communities during outreach to remind mothers about due vaccinations.

Currently, immunization cards are used as a systemized way to record and track vaccinations. However, since cardholder prevalence is < 70% in 21 of 33 least developed countries [14] these cards provide an inaccurate coverage estimate and fail to empower mothers who are unable to read and understand the cards. With the recent proliferation of technology and mobile phones, mHealth, particularly digital registries and SMS reminders provide a promising avenue to improve vaccination coverage. However, evidence for such interventions is mixed and limited [15], particularly when taking into account the fact that the majority of unvaccinated children are concentrated in a small number of countries which do not always have the necessary preconditions for mHealth interventions to succeed [16]. Evidence from studies in Pakistan show low response rates to vaccine oriented SMS reminders as well as no impact on self-reported medication adherence for TB. Some of the main barriers limiting the efficacy of mHealth interventions include inability of caregivers to read the messages [17], frequent changing of phone numbers [17, 18] as well as low phone ownership [19] and literacy rates among women who are most often the primary caretakers and responsible for bringing children for immunization. As an alternate to both paper based and technology based reminders, silicone vaccine reminder bracelets are relatively simple and cheap to use without the need for complicated infrastructure and resources. They make use of numbers and symbols to convey the vaccination schedule and immunization status of the child and therefore have the unique benefit of being suitable for illiterate or less educated mothers. Additionally, they can be adapted to most local settings regardless of the spoken language and infrastructure availability; input from vaccinators and mothers can be used to customize the bracelets to meet the needs and preferences of the communities they are introduced in, giving them an advantage over other home-based reminders. Furthermore, these bracelets are simple to use, inexpensive, and baby-safe.

This study aims to rigorously examine the impact of vaccine reminder bracelets on vaccine schedule adherence in a developing country setting, and validate their effectiveness. Two types of bracelets will be evaluated in this study: One developed by Alma Sana Inc., in 2009, and the other a simple silicone bracelet used by Interactive Research and Development (IRD) in a previous project.

### Study objectives

#### Objective 1: Re-design and adapt bracelets

We will conduct a formative study with both mothers and vaccinators through short interviews to inform the redesign of Alma Sana’s bracelet. The Alma Sana bracelet was initially designed for Peru and Ecuador and prior to implementation in Pakistan, the design of the bracelet will have to be adapted to the local context. This will include changes to the color of the bracelet, the symbols used and most importantly, adapting the vaccination schedule on the bracelet to represent the EPI vaccination schedule followed in Pakistan. Questions will therefore focus on preferences for color, size, culturally relevant or meaningful symbols and any other adaptations that would make the bracelet more appealing. Bracelets will then be manufactured accordingly.

#### Objective 2: Evaluate the impact of Alma Sana bracelet in improving immunization coverage and timeliness of Pentavalent-3/PCV-3/Polio-3 and Measles 1 vaccine in intervention versus control arm in Pakistani children under 2 years of age through a randomized control trial

We will conduct an individually randomized, three-arm parallel group design randomized control trial with equal allocation to evaluate the impact of the Alma Sana bracelet. Children in intervention Group A will receive the adapted Alma Sana bracelet, while children in the control group will not receive any bracelets. To evaluate the bracelets’ impact, we will compare our variables of interest (coverage and timeliness rates of (Pentavalent-3/PCV-3/Polio-3 and Measles 1) between Group A and the Control Group.

#### Objective 3: Evaluate the impact of simple silicone bracelet with symbols in improving immunization coverage and timeliness of Pentavalent-3/PCV-3/Polio-3 and Measles 1 vaccine in intervention versus control arm in Pakistani children under 2 years of age through a randomized control trial

We will conduct an individually randomized, three-arm parallel group design randomized control trial with equal allocation to evaluate the impact of the simple silicon bracelet with symbols. Children in intervention Group B will receive the simple silicone bracelet, while children in the control group will not receive any bracelets. To evaluate the bracelets’ impact, we will compare our variables of interest (coverage and timeliness rates of (Pentavalent-3/PCV-3/Polio-3 and Measles 1) between Group B and the Control Group.

### Secondary objective: Compare the immunization coverage and timeliness rates of Pentavalent-3/PCV-3/Polio-3 and Measles 1 vaccine between the Alma Sana and simple silicone bracelets

We will conduct an individually randomized, three-arm parallel group design randomized control trial with equal allocation to compare the immunization coverage and timeliness rates of Pentavalent-3/PCV-3/Polio-3 and Measles-1 between Intervention Group A (given the Alma Sana bracelets) and Intervention Group B (given the simple silicone bracelets). We will compare the efficacy of the two bracelets’ by doing an inter-arm comparison of our variables of interest between Group A and Group B.

## Methods

### Aim

The overall aim of the intervention is improvement in both immunization coverage rates and timeliness among the study participants through introducing simple silicone bracelets as childhood vaccination reminders and trackers.

### Trial design

**Fig. 1:**
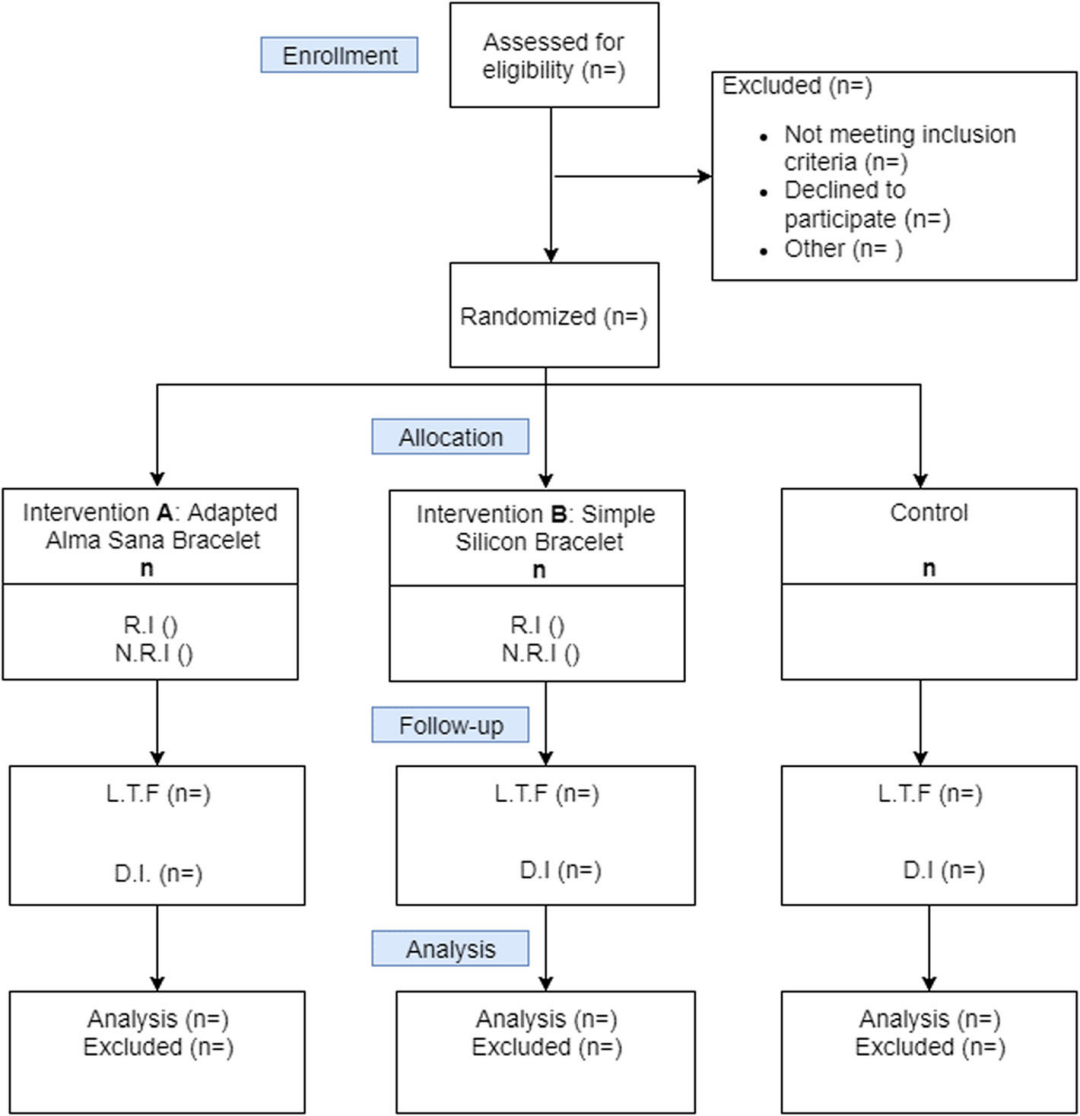
Flow of participants through the study

This will be an individually randomized, three-arm parallel-group trial. The three groups will be equally allocated on a 1:1:1 ratio into two treatment groups and a control group. Children enrolled in the two intervention groups will receive the two different types of bracelets whereas children in the control group will receive no intervention beyond standard care. Figure 1 below shows the study flow.

### Study site and setting

The study will be conducted in 4 Expanded Program on Immunization (EPI) centers located in Landhi Town, Karachi (list of study sites can be obtained from https://clinicaltrials.gov/ct2/show/study/NCT033107620. Centers will be selected based upon the volume of incoming children. Landhi Town is a peri-urban area in the south of Karachi city, in Pakistan’s Sindh province. Karachi is among the 3 districts in Sindh where polio cases have been detected in 2016; within Karachi, Landhi Town is among the highest risk areas for polio prevalence. Landhi Town has an overall estimated population of 872,000 and an annual birth cohort of 30,514 children. The study introduces an intervention designed primarily for illiterate mothers. Landhi Town provides a feasible setting to test the bracelets since the prospective study participants encompass a lower economic stratum, living on $1.25 per day, have low levels of educational attainment, and live in difficult-to-reach areas.

### Participants

Children (male and female) who are 0–3 months old at enrolment, reside in the study catchment area and visit the clinic for BCG or Pentavalent-1/Polio-1/PCV-1 vaccination will be approached for screening to participate in the study.

### Vaccination schedule in Pakistan

The vaccines will be administered as per routine EPI program in Pakistan which include BCG (Bacille Calmette-Guérin) at 0–6 weeks of age, three doses of pentavalent (DPT, HepB, Hib) vaccine, two doses of pneumococcal vaccine (PCV) and three doses of oral polio vaccine at 6, 10 and/or 14 weeks of age, and two doses of measles at 9 and 15 months of age.

### Inclusion and exclusion criteria

Inclusion criteria for the study include children who are (1) presenting to a participating center to receive the BCG or Pentavalent-1/Polio-1/PCV-1 vaccine; (2) accompanied by a primary caregiver; (3) healthy; (4) and (caregivers) have been residing in the study site for more than 6 months. Exclusion criteria include children (1) who are older than 3 months and; (2) whose caregivers plan to go to a different center for the child’s remaining vaccination visits. The study does not involve any vulnerable populations.

### Duration

The study will be conducted over 18 months. Immunization data of all enrolled children will be collected at each vaccination visit following his/her enrolment i.e., when s/he visits for Pentavalent-1 vaccine (if enrolled at BCG) at approximately 6 weeks of age, Pentavalent-2 vaccine at approximately 10 weeks of age, Pentavalent-3 vaccine at approximately 14 weeks of age and Measles 1 vaccine at approximately 9 months old. Participation will continue till the Measles 1 vaccine is administered (recommended age is 9 months), or till eleven months have elapsed since child’s enrolment.

### Sample size

A sample of 1062 participants (354 in each arm) would give 80% power (alpha = 0.05, 2-tailed) to detect a difference of 10% in Pentavalent-3/PCV-3/Polio-3 vaccine coverage rate over the baseline expected coverage rate of 60%. For the same statistical parameters, a sample size of 1155 participants (385 in each arm) would detect a difference of 10% in Measles-1 vaccine coverage rate over the baseline expected coverage of 50%. A sample size of 1155 would give enough power to realise both objectives. The final sample size of 1446 infants (482 in each arm) was selected to allow for a potential dropout rate of 20% during the follow-up period.

### Recruitment and screening

Eligible Children (as identified through the eligibility criteria in the screening form) will be recruited at the participating immunization centers when they arrive for the BCG or Penta-1/PCV-1/OPV-1 vaccine as part of standard of care. Our study staff will be based at the centers to identify these children when they arrive at the centre. Our field workers will obtain consent for screening and then administer a short screening form to determine eligibility. The caregiver accompanying the child for the visit will provide information.

The entire study duration is 18 months and includes planning and actual field implementation. We expect to enroll study participants over a duration of approximately three to four months and follow up with them over the next 11 months. We have allocated approximately four months for enrolling/recruiting participants in the study. Preliminary data from our previous work in the study site indicates that on average there are 100 visits for BCG and Pentavalent-1/Polio-1/PCV-1 vaccine per center per month. Since we will be including 4 centers in our study, we would be able to recruit 1446 participants over a period of approximately three to four months.

### Informed consent

Among those children who are eligible for the study, their caregivers will be approached for enrolment into the study with a consent form informing them of the nature, rationale and anticipated risks and benefits of the study. The consent form will be in the local language and read out to the participants; those who agree to participate will be asked to sign/put their thumbprint on the form to signify their consent. They will be provided time to ask questions and think about their decision to participate during the process of obtaining consent. If the respondent agrees to participate in the study, he/she will be given an instruction card (post randomization) outlining the procedure to be followed in the (treatment or control) group that they have been allocated to, and will also be provided contact details for the relevant person to whom further questions can be directed. Subjects will not receive a copy of the consent form and no audio or video materials will be used during the consent process. At each follow-up visit, the field workers will ensure that participants understand and follow the protocols of the research.

### Participant withdrawal

In the event of participant withdrawal from the study due to withdrawing consent, the date and reason for withdrawal will be properly documented. If the participant was in either of the two treatment groups, they will be asked to return the bracelet. Data collected on participants before withdrawal will be retained but not included in the final analysis. Since this is a minimal risk study, we do not expect any discontinuation of interventions in response to harm caused by the bracelets.

### Randomization

The randomization sequence will be generated by the PI in Stata version 13 with a 1:1:1 allocation, using random block sizes of 3, 6, 9 and 12. The allocation sequence will be concealed from the study staff responsible for screening and enrolling participants in sequentially numbered, opaque, sealed envelopes. After consent and randomization by the field staff, each child will be assigned a unique study ID (a sticker containing the study ID will be pasted on the child’s government issued EPI card). The field staff will sequentially enroll 1446 children from the four participating EPI centers over a period of approximately 3–4 months. Because of the nature of the intervention, neither the participants, nor the field staff enrolling the participants will be blinded during and post randomization.

### Interventions

#### Intervention a; Alma Sana bracelet

**Fig. 2:**
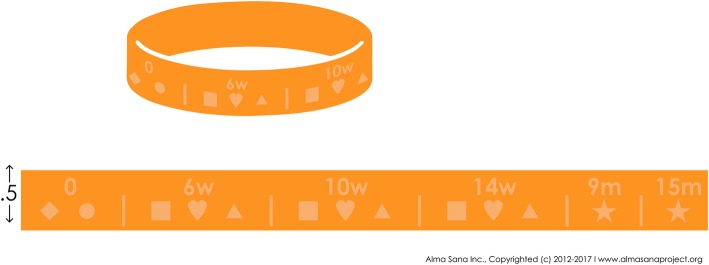
Adapted version of Alma Sana Bracelet (for Pakistan)

A vaccine reminder and tracker bracelet developed by Alma Sana Inc., a 501(c)3 non-profit organization founded in Indianapolis, Indiana, US, will be adapted for use in Pakistan (based on feedback received during the formative phase of the study). Children in intervention Group A will receive these bracelets. The bracelet uses a combination of symbols and numbers to denote the entire vaccine schedule a child is supposed to receive before the age of 2 years. Shapes indicate vaccines, and numbers signify the child’s age (Fig. 2). Additionally, mothers will be provided instructions on how to decode and use the bracelet. Every time a child receives a vaccine, vaccinators will use hole punchers to perforate shapes denoting the particular vaccine a child has received. Mothers can, therefore, look at the bracelet and know which vaccines her child has received and the dates of future visits. The bracelets will be manufactured in 2 different sizes to ensure they fit the child’s wrist as he/she grows older.

#### Intervention B; simple silicone bracelet

**Fig. 3:**
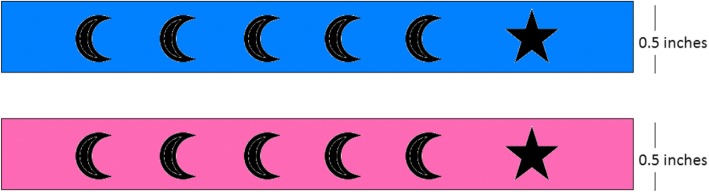
Simple Silicone Bracelets

This is a simple silicone bracelet used by Interactive Research and Development in a previous project. Children in intervention Group B will receive these bracelets. This bracelet has six symbols to remind parents that their child needs to make six distinct vaccination visits before he reaches 2 years of age. The first five symbols are represented by a crescent shape and the sixth symbol is represented by a star shape to denote that the child is fully immunized (Fig. 3). At the time of enrolment, mothers will be provided instructions on how to decode and use the bracelet. Every time a child receives a vaccine, vaccinators will use hole punchers to perforate shapes denoting the particular vaccine a child has received. These bracelets will be manufactured in 2 different sizes and colors (pink for baby girls and blue for baby boys).

All participants (irrespective of whether they are in the intervention group A or B or control group) will receive the routine EPI vaccinations as per Pakistan’s EPI Immunization schedule (one dose of BCG and Polio soon after birth, 3 doses of Pentavalent/Polio/PCV at 6, 10 and 14 weeks, and 2 doses of Measles vaccine and 9 and 15 months of age) and the vaccinator will record the child’s immunization data in the EPI immunization card as well as EPI register. The difference between the intervention and standard care is only the provision of bracelets. The comparator group will only receive the standard care.

#### Improving adherence

To ensure that participants adhere to the interventions, the study team will ensure at the time of enrolment and at each follow-up visit, that participants are aware of the purpose of the bracelet, how to use it and the importance of making their child wear it at all times. Additionally, at each follow up visit, the participants will be observed for whether their child is wearing the bracelet or not and will be asked if they have any questions regarding the bracelet.

### Outcomes

The key study outcomes and their associated measurement is outlined in Table 1 below.

**Table 1:** Outcome Indicators and Measurement

Outcome of Interest	Measured by
At least 10% increase in Pentavalent-3/PCV-3/Polio-3, Measles-1 coverage. Indicators:	Enrolment and follow-up immunization visits.
1. % children vaccinated (on time), by antigen	
2. % of children fully vaccinated	
3. Drop-out rate	
Improved understanding of bracelet use:	Detailed feedback questionnaire
1. Reasons for delayed immunizations	
2. Reasons for dropping out	
3. Do bracelets influence the decision of parents to visit immunization centers on time?	

### Data collection methods

At the child’s first visit to the center, the field worker will use an Enrolment Form to note the name and age of the child along with the immunizations received during the visit in addition to basic demographic information.

For the follow-up visits by the caregivers to get the subsequent vaccinations (3–4 visits), our field workers will be based at the participating EPI centers throughout the follow-up period. The field workers will use the unique study IDs to identify study participants when they come for follow-up visits. At each follow-up visit, the field worker will record the vaccine administered, and date of administration for each child enrolled in the study, including verifying data on past immunizations and due vaccines. Therefore, for each study participant, the field workers will collect data 3–4 times during the follow-up period.

After the completion of the follow-up phase, short (15–20 min) phone or in-person interviews will be conducted to collect follow up vaccination data (if missing) and data on experiences of study participants regarding the bracelets. Dropout from the vaccination schedule is also an outcome. We will be able to identify the caregivers who did not show up for the follow up phase, these participants may be contacted through phone or in person visits towards the end of the follow up period. Key data points to be collected will include reasons for drop-outs, whether mothers understood how to use the bracelets, how often mothers said their babies wore the bracelets and why, what mothers liked and disliked about the bracelets and whether mothers would continue using the bracelets as reminders moving forward.

At all points of the study, data will be collected directly from participants using paper-based questionnaires. Immunization data is copied from the child’s government-issued EPI card. All data will be transferred to a secure electronic medium on a daily basis.

### Data management

Data will be entered and stored in computerized databases maintained under password protection. After the completion of data collection, data will be cleaned and de-identified for analysis. To minimize risk from disclosure, the following mechanisms will be instated: Data collected on paper forms at the EPI center will be handed over to the data entry operator on the same day. Data will be transferred to electronic medium on a regular basis while the paper forms will be stored under lock for reference after being scanned and securely stored on an encrypted hard drive. All data collected and obtained through this study will be property of IRD. After the completion of data collection, data will be cleaned and de-identified for analysis and for sharing with PIs at collaborating centers. A secure file transfer server will be used to transmit the data and where possible, transmission of data through email will be avoided.

### Data confidentiality

For all data collected and obtained through this study, the PI and Program Manager responsible for ensuring confidentiality. The respondents’ identity and information collected will be kept confidential through assigning them unique study IDs. Data will be kept in electronic format on password secured computers at IRD for three years after the study has been completed.

### Data monitoring

Regulatory compliance will be monitored by both the Principal Investigator and the management team through random on-site visits to the immunization centers. They will ensure that the research protocols are being adhered to, and administration of consent and questionnaires is being correctly performed. All documentation and record keeping will be done on a daily basis through monitoring sheets recording the number of forms (screening, consent and data collection) administered by each field worker. Where needed, hard copies of all forms will be scanned, and a soft copy will be retained for the record. Refresher training on protocol adherence and record keeping will be delivered by the Program Manager at least twice during the course of the study. There is no formal data monitoring committee for this trial as this is a minimal risk study with a relatively shorter duration.

### Statistical methods

The baseline characteristics of the three groups will be compared by using the Student’s t-test for continuous variables and the chi-square test for categorical variables (using alpha = 0.05 for evaluating statistical significance). We will use intention to treat analysis—that is, all subjects will be analyzed with the assumption that they remained in the treatment group to which they were initially assigned.

$$ \mathsf{Y}=\mathsf{c}+\mathsf{i}.\left(\mathsf{allocation}\ \mathsf{group}\right)+\mathsf{\gamma X}+\mathsf{\varepsilon} $$

Y is the relevant outcome measure (for instance Pentavalent-3 coverage)

C is the constant that gives the value of the outcome for the control group

Allocation group is a categorical variable with 3 levels; “0-Control Group”, “1-Alma Sana Group”, “2-Simple Silicon Group”.

X is the set of possible covariates (gender, mother’s education etc.)

We will use the xi command to create these indicator variables and run the regression/model all in one command on STATA. Logistic regression will be used to determine whether the difference in outcome between the two treatment groups and control group are statistically significant and whether the difference in outcome between the two treatment groups is statistically significant. Multiple imputation will be used to handle missing data. The data will be analyzed in STATA version 15 (StataCorp. 2017. Stata Statistical Software: Release 15. College Station, TX: StataCorp LLC).

### Ethics

This is a minimal risk study, with no possibility of harm to participants beyond the risks associated with standard care. If any adverse event does occur, field staff is instructed to outline any reportable events through an incident report (to be filled either by the staff or study participant) which must be handed over to the Program manager within 24 h of the event occurrence. The report will be filed and the Program manager will review the report and carry out further investigation if necessary, before reporting to Institutional Review Board (IRB) and the donor. Any unanticipated problems and unexpected Serious Adverse Events (SAE) related to study participation will be communicated by study team within 5 business days of the study team becoming aware of the SAE.

To maintain participant privacy, the outlined provisions for data management and monitoring will be adhered to as much as possible. Participants will only be asked questions that are pertinent to the study and wherever possible will be surveyed separately from other caregivers at the study site. For all data collection activities, participants have the right to discontinue the interview at any time or decline to respond to any question.

Ethical approvals for the study were obtained by Institutional Review Boards of Harvard University and Interactive Research and Development.

### Benefits and costs

There may be a direct benefit to the parent/guardian of study participants in the form of improved immunization coverage through the bracelets. The community may also benefit from information generated as part of the study that could help guide future health and development projects (such as vaccine programs to prevent the target diseases) and health policies with regard to immunizations. Participants will not receive any direct incentive/compensation for participating in the study.

The participants will not encounter any additional costs from participating in the study. The extra time for parents at enrolment will be about 15–20 min at the BCG/Penta1 vaccination visit. No payment/compensation will be provided to any of the study participants.

### Protocol modifications

Any potential changes to the protocol will be agreed upon by PIs and subsequently reported to both IRBs for approval before being conveyed to the study team. Trial records on relevant registries will be updated once ethical approvals are obtained.

### Dissemination strategy

We aim to share the results of the study with the local community including policy makers as well as academia through informal dissemination meetings to discuss our progress, preliminary outcomes, and trends. If feasible, we will include community representatives to share their experience. We will also prepare policy briefs highlighting key research findings with actionable recommendations along with publishing final results of the study in relevant journals. Authorship will be decided according to the ICMJE guidelines. Full protocol will be published in relevant journals and a WHO accredited trial registry.

## Results

Enrolment for the study commenced on July 19, 2017. Each enrolled child will be followed up till the Measles 1 vaccine is administered, or till eleven months have elapsed since enrolment. Proposed duration of the study is 18 months, and expected end date is December 1, 2018. We anticipate that results will be published by January, 2019.

## Discussion

Our expectation is that providing simple to use and easy to understand vaccine reminder bracelets will enable parents to closely keep track and remind themselves of their child’s due vaccines leading to improved coverage and timelines of routine immunizations. Traditionally, to address the global challenge of parents not showing up for vaccination visits [20], low and middle income countries have relied on “Reminder/recall” (RR) interventions including telephone calls, postcards, letters, immunization cards and text messages as a mechanism to remind parents and help in keeping track of children’s vaccination status [21] The standard ‘RR’ protocol utilizes government-issued, paper-based immunization cards. Despite their potential as effective tools for improving vaccination coverage, research into their use has indicated that cards are underutilized and improperly used by caregivers and vaccinators due to lack of literacy among parents and insufficient training of vaccinators in filling out the cards [14]. This results in cards that are illegible, incomplete, or cards that caregivers cannot read or do not value [22]. The most common alternatives to immunization cards that have proven to be effective are phone-based reminders [23, 24]. However, these are resource-intensive at scale and have an uneven impact across the socioeconomic spectrum. A randomized controlled trial in Kenya in 2014 assessing how text-message reminders affected childhood immunization dropout rates saw a 13% reduction in dropouts but also concluded that the intervention had not mitigated the association between a mother’s lack of education and the heightened risk of dropout [25]. Current interventions are, therefore, not optimal solutions in settings with financial and human resource constraints, lack of reliable immunization data on which to base reminders as well as poor coordination between parents and healthcare providers [26].

Wearable immunization reminders have gained prominence in recent years, and are increasingly being recognized as a powerful tool that can improve vaccine adherence in the developing world. Innovations like digital pendants [27], Vaccine Indicator Reminder (VIR) bands [28], beaded bracelets [29] and tattooed bracelets [30] have also been developed. However, beyond feasibility testing, their use and impact have not been investigated, and no concrete evidence exists of their effectiveness at improving immunization outcomes. Moreover, their use remains sporadic and limited to small communities in only a handful of countries.

We recognize a potential limitation of our study stemming from the fact that we are only enrolling children who show up at clinics for immunization and not those who are not vaccinating in the first place. Our objective was to evaluate the direct impact of the bracelets on children who were showing up at clinics. We do acknowledge that the bracelets may have an impact on never vaccinated children due to an externality. However, it is beyond the scope of this study to evaluate this indirect impact and hence enrolment was only confined within the clinic setting.

This study constitutes one of the first attempts to rigorously evaluate an innovative vaccine reminder bracelet. If proven successful, the bracelets will empower low-income caregivers who would otherwise fail to understand and comply with their child’s vaccination schedule. From a financial perspective, by virtue of being low cost, and independent of complex supply chains and technological systems, the bracelets can be integrated into existing immunization setups in any setting, regardless of literacy levels, remoteness of location and technological backwardness. It is expected that results from this study will put forward evidence for an innovative method to improve timely routine immunization coverage in developing countries.

As an additional note, the authors would like to state that the study was on-going at the time of the first submission of this study protocol for publication.

## Data Availability

The datasets generated during the current study are not publicly available since participant follow-ups are on-going.

## Abbreviations

BCG: Bacille Calmette-Guérin
EPI: Expanded Program on Immunization
ICJME: International Committee of Medical Journal Editors
PCV: Pneumococcal conjugate vaccine
